# Rumen-protected guanidinoacetic acid is associated with improved growth performance and altered portal nutrient and hepatic IGF-1 indices in Hu sheep

**DOI:** 10.3389/fvets.2026.1832720

**Published:** 2026-05-04

**Authors:** Wenshu Zhu, Congcong Tian, Wenshuai Zeng, Yong Chen, Mengjian Liu

**Affiliations:** 1 College of Animal Science, Xinjiang Agriculture University, Urumqi, Xinjiang, China; 2 Research Center for Biofeed and Animal Gut Health, Urumqi, Xinjiang, China; 3 Xinjiang Herbivore Nutrition Laboratory for Meat and Milk, Urumqi, Xinjiang, China

**Keywords:** gut–liver axis, Hu sheep, insulin-like growth factor-1 (IGF-1), portal nutrients, RPGAA

## Abstract

**Introduction:**

Guanidinoacetic acid (GAA), the direct precursor of creatine, has been shown to improve growth performance in monogastric animals, but its metabolic effects in ruminants remain unclear. This study examined whether dietary GAA and rumen-protected GAA (RPGAA) are associated with changes in intestinal creatine-related metabolism, portal nutrient concentrations, and hepatic IGF-1 expression in Hu sheep.

**Methods:**

Twenty-seven Hu rams were randomly assigned to three treatments for 45 days: a basal diet (control), the basal diet supplemented with 1.0 g/kg GAA, or the basal diet supplemented with 1.0 g/kg RPGAA. Growth performance, intestinal mucosal GAA and creatine concentrations, expression of genes related to creatine metabolism and nutrient transport, portal blood nutrient concentrations, serum biochemical variables, and hepatic IGF-1 indices were determined.

**Results:**

RPGAA significantly increased final body weight and average daily gain and decreased feed-to-gain ratio (*p* < 0.05). RPGAA increased GAA and creatine concentrations in the jejunal mucosa, downregulated AGAT mRNA expression, and upregulated transporter-related genes (*p* < 0.01). These changes were accompanied by higher concentrations of glucose, arginine, methionine, and lysine in portal blood (*p* < 0.05), as well as increased hepatic IGF-1 mRNA expression and IGF-1 concentration (*p* < 0.01). In addition, serum glucose and albumin increased, whereas total cholesterol and bilirubin decreased in the RPGAA group (*p* < 0.05).

**Discussion:**

These findings suggest that the improved growth performance induced by RPGAA is associated with enhanced jejunal GAA availability, portal nutrient concentrations, and hepatic IGF-1 expression in Hu sheep.

## 1 Introduction

Improving nutrient utilization and growth efficiency remains an important objective in ruminant production (1). Creatine (Cr) plays an important role in cellular energy metabolism by buffering ATP through the phosphocreatine system, thereby supporting muscle growth and metabolic efficiency (2, 3). Guanidinoacetic acid (GAA), the direct precursor of creatine, has received increasing attention as a functional feed additive because it may increase tissue creatine availability and spare arginine for other metabolic functions (4).

In monogastric species, dietary GAA supplementation has been reported to improve growth performance, feed efficiency, and muscle-related traits (5). In ruminants, however, the response to GAA is less consistent because unprotected GAA may be degraded in the rumen before reaching the small intestine (6). Rumen-protected GAA (RPGAA) may therefore be a more effective strategy for increasing the post-ruminal supply of GAA. In ruminants, the small intestine is the primary site for post-ruminal absorption of nutrients and functional additives. Unprotected GAA is easily degraded in the rumen, making intestinal absorption efficiency critical for its bioactivity.

Although the liver and kidneys are considered the major organs involved in creatine metabolism, the small intestine may also contribute to GAA absorption and local metabolic regulation through transporter expression. In addition, increased intestinal nutrient transport may be reflected in portal nutrient concentrations and thereby influence hepatic growth-related pathways, including insulin-like growth factor-1 (IGF-1) synthesis. However, in ruminants, the intestinal site of GAA absorption and its relationship with portal nutrient availability and hepatic IGF-1 expression remain poorly defined.

We hypothesized that dietary GAA, especially RPGAA, is related to increased intestinal GAA bioavailability, portal nutrient concentrations, and hepatic IGF-1 expression, which are associated with improved growth performance in Hu sheep.

## 2 Materials and methods

### 2.1 Experimental materials and location

The experiment was conducted at a commercial sheep farm in Xinjiang, China, from June to September 2024. During the trial, ambient temperature ranged approximately from 15 to 35 °C.

### 2.2 Supplements

The GAA and rumen-protected GAA (RPGAA) used in this study were provided by Hebei Guangrui Biotechnology Co., Ltd. (Shijiazhuang, Hebei, China; purity ≥98.5%). RPGAA was a coated preparation designed to reduce ruminal degradation. According to a batch-specific quality-control report provided by the manufacturer, a Menke-style *in vitro* rumen simulation test showed that the 12-h ruminal degradation rate of RPGAA was ≤15%, whereas that of uncoated GAA exceeded 90%. These data support reduced ruminal disappearance of the coated product under *in vitro* conditions. However, batch-specific intestinal release data were not available in the present study; therefore, the intestinal release efficiency of this product was not directly quantified.

### 2.3 Animal care and ethical considerations

All experimental protocols were approved by the Institutional Animal Care and Use Committee (IACUC) of the College of Veterinary Medicine, Xinjiang Agricultural University, and conducted in strict accordance with ethical guidelines. The study was reported in accordance with the ARRIVE guidelines.

### 2.4 Animals, diets, and feeding regimen

Twenty-seven healthy male Hu sheep (4–5 months of age; initial body weight 24.15 ± 2.06 kg) were randomly assigned to three dietary treatments (*n* = 9 per treatment). The treatments were: (1) Control, basal diet; (2) GAA, basal diet supplemented with 1.0 g/kg GAA (DM basis); and (3) RPGAA, basal diet supplemented with 1.0 g/kg RPGAA (DM basis). The basal diet was formulated according to NRC (2007) requirements (Table 1).

**Table 1:** Composition and nutritional levels of basic feed (DM basis).

Items	Content (%)	Items	Content (%)
Ingredients		Nutrient levels	
Yellow corn	30.00	Digestible energy DE/(MJ/kg)	8.51
Wheat bran	7.20	Crude protein (CP)	16.45
Soybean meal (CP 44%)	12.00	Ether extract (EE)	1.63
Cottonseed meal (CP 43%)	7.80	Neutral Detergent Fiber (NDF)	45.16
Premix	3.00	Acid Detergent Fiber (ADF)	33.62
Alfalfa hay	20.00	Ash	8.40
Wheat straw	20.00	Calcium	0.59
		Phosphorus	0.32
		Lysine	1.52
		Methionine + Cysteine	0.96
Total	100.00		

(1) Without adding any antibiotic to the feed ingredients. (2) The premix provided the following per kg of feed: VA 8,000 IU, VB 9 mg, VB_2_ 1.1 mg, VB_5_ 2.4 mg, VB_6_ 1.3 mg, VB_12_ 0.01 mg, VD_3_ 500 IU, VE 10 IU, Fe 20 mg, Mn 10 mg, Cu 4 mg, Zn 20 mg, I 0.1 mg, Se 0.1 mg. (3) Crude protein, ADF, NDF, EE and crude ash are measured values, while other nutrient levels are calculated values.

### 2.5 Feeding management

Sheep were housed under the same management conditions and had free access to water throughout the experiment. Diets were offered twice daily at 08:00 and 18:00. Feed offered and refusals were recorded daily to calculate average daily feed intake (ADFI), and body weight was measured at the beginning and end of the trial to calculate average daily gain (ADG) and feed-to-gain ratio (F/G). Pen was considered the experimental unit for intake-related variables, whereas the animal was the experimental unit for blood and tissue measurements.

### 2.6 Sample collection

#### 2.6.1 Jugular venous blood samples

One day before trial completion, 5 mL of jugular venous blood was collected with a disposable needle, centrifuged at 3,500 rpm for 15 min at room temperature, and serum was aliquoted into 1.5 mL tubes and stored at −20 °C for biochemical analyses.

### 2.7 Portal vein blood collection

At the end of the experiment, sheep were fasted overnight and then slaughtered according to standard commercial procedures. After stunning and abdominal opening, portal venous blood was collected immediately using heparinized tubes before complete exsanguination. Samples were centrifuged at 3,000 × g for 15 min at 4 °C, and plasma was stored at −80 °C until analysis. Intestinal mucosa and liver tissue were then collected, snap-frozen in liquid nitrogen, and stored at −80 °C. Portal blood was sampled after overnight fasting, which may reflect basal metabolism rather than post-prandial absorption flux.

### 2.8 Tissue samples

After slaughter according to commercial procedures, the abdominal cavity was opened for tissue collection. About 10 g of liver tissue was excised, placed into RNase-free cryovials, and immediately frozen in liquid nitrogen. Segments of duodenum, jejunum, and ileum were collected; luminal contents discarded, and the lumens were rinsed three times with sterile physiological saline. After opening longitudinally, residual contents were removed, and the mucosa was rapidly scraped with a glass slide, then homogenized and placed into RNase-free cryovials for subsequent gene expression and enzyme activity assays. Ileal mucosa was collected but not analyzed for GAA, Cr, and CK due to limited sample throughput and focus on the primary absorptive segments.

### 2.9 GAA and Cr contents in small intestinal mucosa

Approximately 0.1 g of frozen small intestinal mucosal tissue was accurately weighed and placed into pre-cooled centrifuge tubes. Corresponding extraction buffers were added according to the requirements for each target analyte: for GAA analysis, 1.0 mL of a disodium hydrogen phosphate buffer-acetonitrile mixture (73:27, v/v); for creatine (Cr) analysis, 1.0 mL of 5% trichloroacetic acid; for CK analysis, pre-cooled PBS buffer (pH 7.4) was added at a tissue mass (g) to PBS volume (mL) ratio of 1:9. The samples were homogenized using a tissue homogenizer for 3 min until a homogenate state was achieved, followed by ultrasonic lysis on ice for 10 min to obtain the extraction homogenate for subsequent analysis.

GAA content was determined using high-performance liquid chromatography (HPLC; LC-20AT, Shimadzu, Kyoto, Japan). The GAA extraction homogenate was vortex-mixed for 5 min, diluted to a final volume of 10 mL, filtered through a 0.22 μm organic phase syringe filter, and the filtrate was collected in a sample vial for analysis. Chromatographic conditions were as follows: column, AQ-C18 (150 mm × 2.1 mm, 3 μm); mobile phase A, pure acetonitrile; mobile phase B, disodium hydrogen phosphate buffer-acetonitrile mixture (73:27, v/v); isocratic elution (A: B = 2:98, v/v); flow rate, 1.0 mL/min; column temperature, 30 °C; detection wavelength, 195 nm; automatic injection with an injection volume of 20 μL.

Cr content was determined using HPLC. The creatine extraction homogenate was vortex-mixed for 5 min, adjusted to neutral pH with 1 mol/L sodium hydroxide solution, diluted to 1.3 mL with 0.02 mol/L potassium dihydrogen phosphate buffer, centrifuged at 4 °C and 10,000 r/min for 10 min, and the supernatant was filtered through a 0.22 μm aqueous phase syringe filter for analysis. Chromatographic conditions were as follows: column, AQ-C18 (250 mm × 4.6 mm, 5 μm); mobile phase A, pure methanol; mobile phase B, 0.02 mol/L potassium phosphate buffer (pH 7.0); isocratic elution (A: B = 3:97, v/v); flow rate, 0.8 mL/min; column temperature, 30 °C; detection wavelength, 210 nm; automatic injection with an injection volume of 10 μL.

### 2.10 Enzyme-linked immunosorbent assay

The concentrations of creatine kinase (CK) in intestinal mucosal homogenates were determined using commercial ELISA kits (Fankewei, China) according to the manufacturer’s instructions. The tissue extraction homogenates were centrifuged at 4 °C and 12,000 r/min for 20 min, and the supernatants were collected as test samples. Commercial ELISA kits (Fankewei, China) were used strictly according to the manufacturer’s instructions. Absorbance was measured at a wavelength of 450 nm using a microplate reader, and the concentrations of CK were calculated from the standard curves.

The assay was performed in strict accordance with the instructions of the IGF-1 ELISA kit (Nanjing Jiancheng Bioengineering Institute, China). After adding standards or samples, the plate was incubated at 37 °C for 2 h. Following washing, a biotinylated primary antibody was added, and the plate was incubated at 37 °C for 1 h. After washing again, HRP-labeled avidin was added, and the plate was incubated at 37 °C for 30 min. TMB substrate solution was added, and the plate was incubated at 37 °C in the dark for 15 min. Finally, stop solution was added. The optical density (OD) was measured at 450 nm using a microplate reader. Results were expressed as ng/mL for portal plasma and as ng/mg protein for liver homogenates.

### 2.11 RNA extraction

Total RNA was extracted from approximately 30 mg of frozen intestinal mucosal tissue using the HiPure RNA Mini Kit (Magen, China) following the manufacturer’s protocol. RNA purity was evaluated by measuring the OD260/OD280 ratio (within 1.8–2.0) using an Infinite M200 microplate reader (Tecan, Männedorf, Switzerland). RNA integrity was confirmed by 1% agarose gel electrophoresis. All RNA samples were stored at −80 °C for subsequent analysis.

### 2.12 Reverse transcription

cDNA was synthesized from total RNA using a reverse transcription kit (Vazyme, China) following the manufacturer’s protocol, and stored at −20 °C for subsequent analysis.

### 2.13 Real-time quantitative PCR

Real-time quantitative PCR (qPCR) was performed using β*-actin* as the internal reference gene. Gene-specific primers (Table 2) were used to determine the relative mRNA expression levels of target genes, including AGAT, GAMT, relevant transporter genes, and IGF-1. Each 10 μL reaction contained ChamQ SYBR qPCR Master Mix (Vazyme, China). Amplification was carried out in triplicate on a thermal cycler (CFX96, Bio-Rad, Hercules, CA, United States), and relative gene expression was calculated using the 2^−ΔΔCt^ method.

**Table 2:** Primer sequences and annealing temperatures of target genes and internal reference gene.

Gene	Prime sequence (5′ → 3′)	Annealing temperatures °C	GenBank number
AGAT	F: AATGGAAGGAGTGACAGTGAGGAG R: CATCGCACCATATAAACCCGTAGAC	58	XM_004010649.5
GAMT	F: CAAACATGGTGGTGATGTCCG R: CAGGGGGTGTCCTCACCTA	57	XM_015096020.4
SLC6A6	F: AAGGTGTCCTCCAGGCAATG R: CATCGTGATCGTGTCCCTCC	58	XM_027957816.3
SLC6A8	F: GGATTCCTCCAATCAGGGCAA R: TCCCCTACCTGTGCTACAAGA	57	XM_027962936.2
IGF-1	F: ATCCTCCTCGCATCTCTTCTATCTG R: GCACACGAACTGGAGAGCATC	58	XM_060411609.1
β-actin	F: CCATCGGCAATGAGCGGTTC R: GGAATTGAAGGTAGTTTCGTGAATGC	58	XM_060405599.1

### 2.14 Glucose and amino acids in portal venous blood

Plasma glucose concentration was measured using a microplate reader (Infinite M200, Switzerland) at 340 nm according to the instructions of the glucose assay kit (Nanjing Jiancheng Bioengineering Institute, Nanjing, China) based on the hexokinase method. The concentration was calculated using a standard curve.

Amino acid concentrations were measured using HPLC (LC-20AT, Shimadzu, Kyoto, Japan). Portal venous plasma samples were pre-processed as follows: 200 μL of plasma was mixed with 400 μL of acetonitrile for protein precipitation and centrifuged at 4 °C and 12,000 r/min for 10 min. Then, 100 μL of the supernatant was taken, mixed with 50 μL of PITC derivatization reagent, and incubated at 55 °C for 20 min. After adding 1 mL of *n*-hexane, the mixture was vortexed thoroughly and allowed to stand for 10 min. The lower aqueous layer was collected, filtered through a 0.22 μm membrane filter, and analyzed. Chromatographic conditions were as follows: column, C18 (4.6 mm × 250 mm, 5 μm; Phenomenex Inc., Torrance, CA, United States); mobile phase A: 0.1 mol/L sodium acetate buffer (pH 6.5)-acetonitrile (93:7, v/v); mobile phase B: acetonitrile-water (80:20, v/v); gradient elution: 0–10 min, A 95–80%; 10–20 min, A 80–50%; 20–30 min, A 50–0%; 30–35 min, A 0%; 35–40 min, A 0–95%; flow rate, 1.0 mL/min; column temperature, 40 °C; detection wavelength, 254 nm; injection volume, 20 μL. For quantitative analysis, a mixed amino acid standard (including arginine, lysine, leucine, isoleucine, valine, methionine, phenylalanine and tryptophan) was prepared and derivatized, and a standard curve was plotted (*R*^2^ ≥ 0.999). The concentration of each amino acid in the samples was calculated based on the corresponding peak area.

### 2.15 Determination of IGF-1 concentration in the liver of Hu sheep

Liver tissue homogenate preparation: Approximately 100 mg of liver tissue was weighed, and 1,000 μL of pre-cooled RIPA lysis buffer (containing 1% PMSF and 1% phosphatase inhibitor) was added. The tissue was homogenized using a high-throughput tissue grinder for 3 min, followed by ice-bath lysis for 30 min. The homogenate was centrifuged at 4 °C and 12,000 r/min for 10 min, and the supernatant was collected. Separately, portal venous plasma samples were thawed at room temperature for use. A protein quantification kit (Beyotime, China) was used according to the manufacturer’s instructions to determine the protein concentration of the liver supernatant and serum samples, and the protein concentration of liver homogenates was adjusted to the same level across samples before ELISA.

### 2.16 Serum biochemical indicators

Serum biochemical variables were measured using an automatic analyzer (7,600, Hitachi, Tokyo, Japan), including blood urea nitrogen (BUN), glucose (GLU), alanine aminotransferase (ALT), aspartate aminotransferase (AST), ALT/AST ratio, total bilirubin (TBIL), alkaline phosphatase (ALP), total protein (TP), albumin (ALB), globulin (GLOB), albumin-to-globulin ratio (A/G), triglyceride (TG), total cholesterol (TC), and creatinine (CRE).

### 2.17 Statistical analysis of data

All experimental data were initially organized and preliminarily processed using EXCEL (version 16.0.2408). Statistical analysis was performed using IBM SPSS Statistics (version 18.0). Data were first tested for normality. Subsequently, one-way analysis of variance (one-way ANOVA) was performed. When treatment effects were significant, means were compared using Tukey’s multiple comparison test.

Results are presented as “mean ± standard error (SE).” Differences were considered statistically significant at *p* < 0.05.

## 3 Results

### 3.1 Effects of GAA and RPGAA supplement on the growth performance of Hu sheep

The effects of dietary supplementation with GAA or RPGAA on the growth performance of Hu sheep are shown in Table 3. Compared with the control group, dietary supplementation with GAA or RPGAA significantly increased FW and ADG (*p* < 0.05). Likewise, the ADFI was significantly higher in both the GAA and RPGAA groups than in the control (*p* < 0.05). Additionally, F/G was significantly lower in both treatment groups relative to the control (*p* < 0.05). These results show that both GAA and RPGAA improved growth performance, with the lowest F/G observed in the RPGAA group.

**Table 3:** Effects of dietary supplementation with GAA or RPGAA on the growth performance of Hu sheep.

Items	Control	GAA	RPGAA	*p*-value
IW/kg	23.44 ± 1.76	24.31 ± 2.24	24.71 ± 2.40	0.531
FW/kg	28.48 ± 0.9^b^	30.83 ± 2.40^a^	32.24 ± 2.13^a^	0.032
ADG/(kg/d)	0.13 ± 0.04^b^	0.16 ± 0.02^a^	0.19 ± 0.02^a^	0.024
ADFI/(kg/d)	1.45 ± 0.12^b^	1.58 ± 0.12^a^	1.61 ± 0.11^a^	0.046
F/G	11.49 ± 0.91^a^	9.71 ± 0.75^b^	8.53 ± 0.58^c^	0.033

IW, Initial weight; FW, Final weight; ADG, Average daily gain; ADFI, Average daily feed intake; F/G, Feed-to-gain ratio. Values in the same row with different lowercase superscript letters indicate significant differences (*p* < 0.05). The same lowercase letter or no superscript letter indicates no significant difference (*p* > 0.05).

### 3.2 Effects of GAA and RPGAA supplementation on GAA, Cr content, and CK enzyme activity in small intestinal mucosa

The effects of dietary GAA or RPGAA supplementation on GAA, Cr, and creatine kinase (CK) content in the small intestinal mucosa of Hu sheep are presented in Table 4. This study evaluated the deposition and metabolic characteristics of GAA and Cr, as well as CK activity, in the mucosa of the mid-duodenum and mid-jejunum. In the duodenal mucosa, GAA and Cr contents, as well as CK activity, exhibited an increasing trend in the GAA and RPGAA groups compared with the control group; however, these differences did not reach statistical significance (*p* > 0.05).

**Table 4:** Effects of dietary GAA or RPGAA supplementation on GAA, Cr, and CK content in the duodenal and jejunal mucosa of Hu sheep.

Intestinal segment	Items	Control	GAA	RPGAA	*P*-value
Duodenum	GAA (μg/g)	48.98 ± 6.84	54.25 ± 6.47	56.06 ± 0.97	0.056
	Cr (μg/g)	596.16 ± 74.57	693.52 ± 75.18	724.92 ± 122.39	0.103
	CK (U/g)	1.24 ± 0.12	1.51 ± 0.22	1.59 ± 0.64	0.565
Jejunum	GAA (μg/g)	11.99 ± 1.28^b^	13.93 ± 1.07^b^	23.99 ± 1.41^a^	0.031
	Cr (μg/g)	541.08 ± 72.48^b^	583.54 ± 96.07^b^	695.65 ± 42.83^a^	0.048
	CK (U/g)	1.19 ± 0.11	1.13 ± 0.18	1.58 ± 0.40	0.062

(1) Data for the ileal mucosa were not measured/included in this analysis. (2) Within the same row, different lowercase superscript letters indicate significant differences (*P* < 0.05), and the same lowercase letters or no letters indicate no significant difference (*P* > 0.05).

In the jejunal mucosa, the RPGAA group showed higher GAA and creatine concentrations than the control and GAA groups (*p* < 0.05), whereas differences in the duodenal mucosa were not significant (*p* > 0.05). No significant difference was observed between the control and GAA groups (*p* > 0.05). No significant differences in CK activity were observed among the groups in the jejunum (*p* > 0.05). Overall, RPGAA increased GAA and creatine concentrations in the jejunal mucosa (*p* < 0.05), whereas no significant differences were observed in the duodenal mucosa (*p* > 0.05).

### 3.3 Effects of GAA and RPGAA supplementation on AGAT, GAMT, and transporter-related gene expression in the small intestinal mucosa

**Figure 1:**
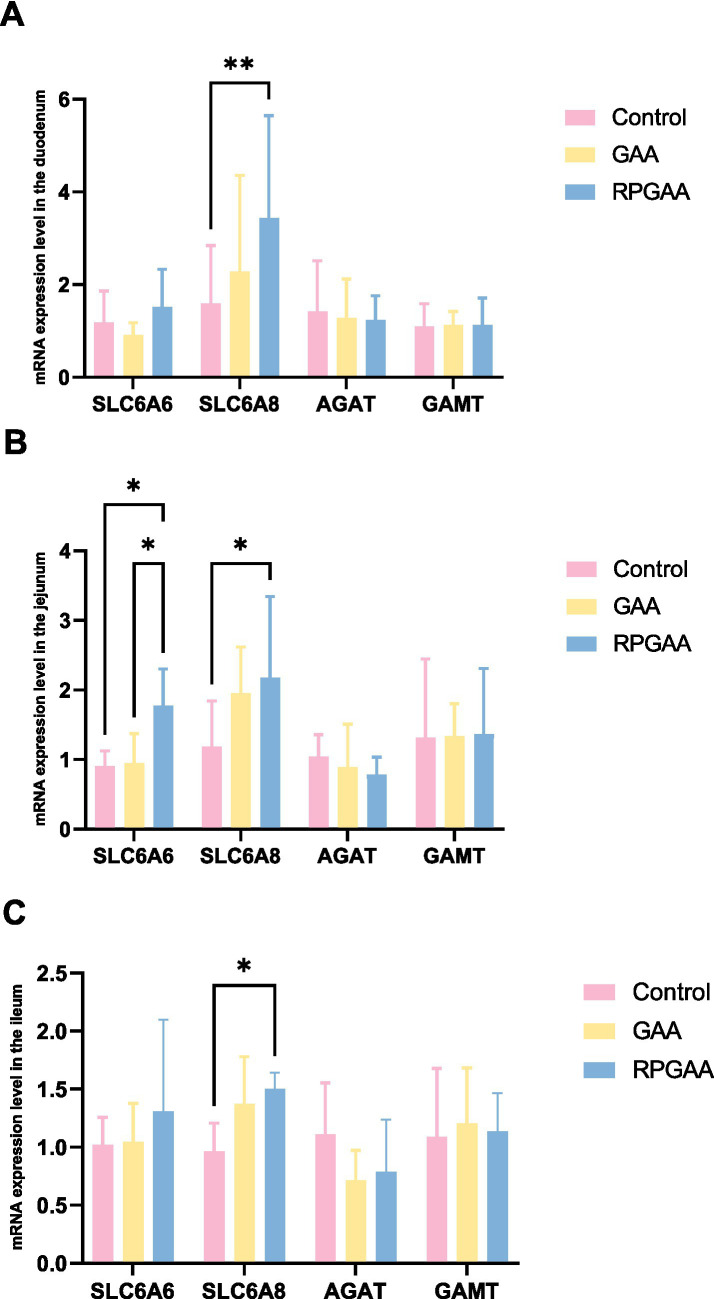
Relative mRNA expression profiles of creatine metabolism-related enzymes and nutrient transporter genes in the **(A)** duodenum, **(B)** jejunum, and **(C)** ileum of Hu sheep in response to guanidinoacetic acid (GAA) and rumen-protected GAA (RPGAA) supplementation. Data are presented as the mean ± standard error (SE). Statistical significance was determined by one–way ANOVA followed by Tukey’s multiple comparison test. Asterisks indicate significant differences compared with the control group: *p < 0.05, **p < 0.01. Error bars represent SE.

Figures 1A–C illustrate the relative expression levels of target genes in the duodenum, jejunum, and ileum, respectively. As shown in Figure 1A, in the duodenal mucosa, the expression level of arginine:glycine amidinotransferase (AGAT) in the RPGAA group was significantly lower than that in the control group, whereas the expression levels of guanidinoacetate N-methyltransferase (GAMT) and solute carrier family 6 member 8 (SLC6A8) were significantly higher (*p* < 0.05). In the GAA group, only SLC6A8 expression levels were significantly elevated compared with the control group (*p* < 0.05).

As shown in Figure 1B, in the jejunal mucosa, AGAT expression levels in both the GAA and RPGAA groups were significantly lower than those in the control group (*p* < 0.05), whereas the expression levels of GAMT, SLC6A8, and SLC6A6 were significantly higher (*p* < 0.05). Moreover, all detected enzymes and transporter-related genes exhibited significant differences between the RPGAA and GAA groups (*p* < 0.05).

As shown in Figure 1C, in the ileal mucosa, the expression levels of SLC6A8 in the RPGAA group were significantly higher than those in the control group (*p* < 0.05).

### 3.4 Effects of GAA and RPGAA supplementation on nutrient concentrations in the portal venous blood of Hu sheep

The effects of dietary GAA or RPGAA supplementation on nutrient concentrations in portal venous blood of Hu sheep are shown in Table 5. As presented in Table 5, dietary supplementation with either GAA or RPGAA selectively modulated nutrient concentrations in the portal venous blood of Hu sheep. Portal glucose concentration displayed a clear gradient, increasing sequentially from the control group to the RPGAA group, with significant differences observed among all groups (*p* < 0.05). The concentration of arginine was significantly elevated in the RPGAA group compared with both the GAA and control groups (*p* < 0.05). Similarly, lysine concentration was significantly greater in the RPGAA group than in the other two groups (*p* < 0.05), with no significant difference between the control and GAA groups (*p* > 0.05). Methionine concentration was higher in the RPGAA group than in the control group (*p* < 0.05), whereas the GAA group was intermediate. In contrast, the concentrations of the essential amino acids leucine, isoleucine, valine, phenylalanine, and tryptophan showed a tendency to increase in the treatment groups, but these changes did not reach statistical significance (*p* > 0.05).

**Table 5:** Effects of dietary GAA or RPGAA supplementation on nutrient concentrations in portal venous blood of Hu sheep.

Items	Control	GAA	RPGAA	*P*-value
Glucose (mmol/L)	4.52 ± 0.24^c^	4.75 ± 0.26^b^	5.18 ± 0.22^a^	0.019
Thr (nmol/L)	20.51 ± 12.72	17.78 ± 11.20	33.00 ± 13.20	0.075
Ser (nmol/L)	22.51 ± 7.79	16.14 ± 10.29	29.51 ± 15.18	0.123
Gly (nmol/L)	36.00 ± 14.51	29.15 ± 22.24	44.00 ± 16.34	0.326
Ala (nmol/L)	43.00 ± 9.59	31.46 ± 23.63	51.00 ± 8.43	0.087
Cys (nmol/L)	36.71 ± 33.76	2.41 ± 3.46	36.08 ± 34.82	0.055
Met (nmol/L)	2.89 ± 0.31 ^b^	8.04 ± 6.23 ^ab^	6.64 ± 0.29 ^a^	0.041
Ile (nmol/L)	50.50 ± 63.30	15.07 ± 9.18	43.50 ± 27.25	0.244
Phe (nmol/L)	17.51 ± 8.34	13.83 ± 11.23	17.50 ± 2.22	0.632
Lys (nmol/L)	37.51 ± 5.75 ^b^	41.50 ± 16.58 ^b^	62.51 ± 27.13 ^a^	0.047
Arg (nmol/L)	8.71 ± 3.70 ^b^	10.39 ± 8.81^ab^	16.79 ± 3.76 ^a^	0.048

Values in the same row with different lowercase superscript letters indicate significant differences (*p* < 0.05). The same lowercase letter or no superscript letter indicates no significant difference (*p* > 0.05).

Some amino acids in portal blood showed high variation, possibly due to individual differences in absorption and fasting status. Overall, RPGAA was associated with higher portal concentrations of glucose, arginine, lysine, and methionine.

### 3.5 Effects of GAA and RPGAA supplementation on IGF-1 levels in the liver and portal venous blood of Hu sheep

The effects of dietary GAA or RPGAA supplementation on IGF-1 related indicators in liver of Hu sheep are shown in Table 6. As shown in Table 6, the concentration of IGF-1 in portal venous blood differed significantly among the groups, with the RPGAA group showing significantly higher levels than the other two groups (*p* < 0.05), and the GAA group also being significantly higher than the control group (*p* < 0.05). Similarly, IGF-1 concentration in liver tissue homogenates exhibited significant intergroup differences, with the RPGAA group being significantly higher than both the control and GAA groups (*p* < 0.05), whereas no significant difference was observed between the control and GAA groups (*p* > 0.05).

**Table 6:** Effects of dietary GAA or RPGAA supplementation on IGF-1 related indicators in liver of Hu sheep.

Items	Control	GAA	RPGAA	*P*-value
IGF-1 concentration in portal venous blood (ng/mL)	34.51 ± 3.26^c^	37.22 ± 4.12^b^	42.35 ± 3.71^a^	0.025
IGF-1 concentration in liver (ng/mg protein)	10.50 ± 1.30^b^	11.42 ± 1.57^b^	16.26 ± 1.82^a^	0.031
Relative mRNA expression of IGF-1 in hepatocytes	1.00 ± 0.13^c^	1.25 ± 0.24^b^	1.73 ± 0.23^a^	0.017

Within the same row, different lowercase superscript letters indicate significant differences (*p* < 0.05), and the same lowercase letters or no letters indicate no significant difference (*p* > 0.05).

At the transcriptional level, relative IGF-1 mRNA expression in hepatocytes displayed a pronounced gradient among the groups, with the RPGAA group showing significantly higher expression than the GAA group (*p* < 0.05), and the GAA group showing significantly higher expression than the Control group (*p* < 0.05). These results show that RPGAA increased hepatic IGF-1 mRNA expression and IGF-1 concentration.

Overall, GAA and RPGAA increased hepatic IGF-1 mRNA expression and IGF-1 concentrations in portal plasma and liver homogenates, with the highest values observed in the RPGAA group.

### 3.6 Effects of GAA and RPGAA supplementation on serum biochemical indicators in venous blood

The effects of dietary GAA or RPGAA supplementation on serum biochemical indicators of Hu sheep are shown in Table 7. Serum biochemical indicators were systematically analyzed to elucidate the overall physiological effects of GAA and RPGAA on Hu sheep from the perspective of systemic metabolism. Serum glucose concentration exhibited a highly significant graded difference among the groups (*p* < 0.001), with the RPGAA group being significantly higher than the GAA group, and the GAA group being significantly higher than the control group. Serum total protein did not differ significantly among groups (*p* > 0.05), whereas albumin increased stepwise from control to GAA to RPGAA (*p* < 0.001). In contrast, Globulin concentration did not differ among groups, whereas the albumin-to-globulin ratio increased significantly in the supplemented groups.

**Table 7:** Effects of dietary GAA or RPGAA supplementation on serum biochemical indicators of Hu sheep.

Items	Control	GAA	RPGAA	*P*-value
BUN/(mmol/L)	1.92 ± 0.03	1.93 ± 0.02	1.94 ± 0.03	0.672
GLU/(mmol/L)	4.65 ± 0.09^c^	4.90 ± 0.09^b^	5.35 ± 0.1^a^	<0.001
ALT/(U/L)	22.88 ± 2.15	21.67 ± 2.81	20.92 ± 1.71	0.438
AST(U/L)	123.67 ± 5.92	117.55 ± 7.71	116.98 ± 1.78	0.125
ALT/AST	0.19 ± 0.03	0.19 ± 0.02	0.18 ± 0.02	0.546
TBIL/(μmol/L)	0.20 ± 0.02^a^	0.15 ± 0.04^b^	0.14 ± 0.04^b^	0.048
ALP/(U/L)	271 ± 10.49^a^	223.33 ± 1.63^b^	227.90 ± 1.53^b^	<0.001
TP/(g/L)	64.18 ± 0.07	64.62 ± 0.19	64.53 ± 0.40	0.470
ALB/(g/L)	29.27 ± 0.70^c^	30.75 ± 0.38^b^	32.32 ± 0.38^a^	<0.001
GLOB/(g/L)	29.67 ± 0.42	29.87 ± 0.55	30.05 ± 0.93	0.369
A/G	0.99 ± 0.03^c^	1.03 ± 0.02^b^	1.08 ± 0.03^a^	0.003
TG/(mmol/L)	0.34 ± 0.03	0.36 ± 0.03	0.36 ± 0.04	0.501
TC/(mmol/L)	2.13 ± 0.11^a^	1.97 ± 0.02^b^	1.97 ± 0.03^b^	0.043
CRE/(μmol/L)	51.67 ± 0.92	51.60 ± 0.70	52.12 ± 0.50	0.712

BUN, Blood Urea Nitrogen; GLU, Glucose; ALT, Alanine Aminotransferase; AST, Aspartate Aminotransferase; ALT/AST, Alanine Aminotransferase/Aspartate Aminotransferase Ratio; TBIL, Total Bilirubin; ALP, Alkaline Phosphatase; TP, Total Protein; ALB, Albumin; GLOB, Globulin; A/G, Albumin/Globulin Ratio; TG, Triglyceride; TC, Total Cholesterol; CRE, Creatinine. Within the same row, different lowercase superscript letters indicate significant differences (*P* < 0.05), and the same lowercase letters or no letters indicate no significant difference (*P* > 0.05).

Among liver function–related indicators, total bilirubin concentration and alkaline phosphatase activity were significantly lower in the GAA and RPGAA groups than in the control group (*p* < 0.05). However, alanine aminotransferase (ALT) activity, aspartate aminotransferase (AST) activity, and the ALT/AST ratio showed no significant differences among the three groups (*p* > 0.05). With respect to lipid metabolism, total cholesterol concentration differed significantly among groups, with both treatment groups exhibiting significantly lower levels than the Control group (*p* < 0.05). In contrast, triglyceride, urea nitrogen, and creatinine concentrations showed no significant differences among the groups (*p* > 0.05) and remained within normal physiological ranges.

Collectively, GAA and RPGAA supplementation was associated with changes in serum indices related to energy and protein metabolism, without adverse changes in the measured liver- or kidney-related biomarkers.

## 4 Discussion

### 4.1 Effects of exogenous GAA on growth performance

The present study showed that dietary GAA supplementation improved growth performance in Hu sheep, with a more pronounced response in the RPGAA group. Compared with the control group, sheep receiving RPGAA had higher ADG and lower F/G over the 45-day period.

These findings are aligned with prior studies in which dietary GAA supplementation was reported to elevate tissue creatine levels and enhance energy metabolism profiles in sheep and Angus cattle (7, 8). Overall, these results support the potential of GAA, particularly in rumen-protected form, to improve growth performance in Hu sheep under the conditions of this study. Because heat-stress-related indicators were not measured, the contribution of ambient temperature to the observed responses cannot be determined (9).

The better effect of RPGAA may be related to reduced ruminal degradation, but this was not verified experimentally (10–12). Rumen protection may increase the proportion of supplemented GAA that reaches the small intestine, which could contribute to the stronger jejunal response observed with RPGAA. Previous studies in cattle have reported effects of RPGAA on carcass and meat-quality traits; however, carcass traits were not evaluated in the present study (13, 14).

### 4.2 Primary intestinal segment and regulation of exogenous GAA absorption and metabolism in the intestine

Cr plays a crucial role in cellular energy metabolism. It is synthesized from its direct precursor, GAA, via the sequential enzymatic reactions catalyzed by AGAT and GAMT (15). Cellular uptake of creatine is mediated primarily by the sodium- and chloride-dependent transporter SLC6A8, while creatine kinase (CK) facilitates rapid ATP regeneration and intracellular energy buffering (16). Importantly, AGAT activity is tightly regulated through negative feedback by creatine and competitive inhibition by ornithine, rendering AGAT the rate-limiting step in endogenous GAA synthesis and providing a strong biochemical rationale for exogenous GAA supplementation to support growth performance (17, 18).

Although the liver and kidneys are traditionally regarded as the principal sites of creatine biosynthesis, previous studies suggest that the intestinal mucosa may also participate in GAA handling and creatine-related metabolism through local expression of AGAT, GAMT, and transporter genes (19, 20). In the present study, GAA and creatine concentrations in the jejunal mucosa were significantly higher in the RPGAA group than in the control and free GAA groups (*p* < 0.05), whereas no significant changes were observed in the duodenum. The higher jejunal GAA and creatine concentrations suggest that the jejunum may be a responsive intestinal segment to RPGAA supplementation under the present experimental conditions (21). The jejunum is the major site for amino acid and peptide absorption with higher expression of SLC6A8 and longer retention time, explaining the stronger response. Compared with the jejunum, the duodenum showed smaller changes in mucosal GAA and creatine, indicating a weaker response in this study (22). Therefore, direct confirmation of segment-specific GAA absorption in sheep will require isotope tracing or intestinal flux measurements.

At the transcriptional level, RPGAA supplementation significantly downregulated jejunal AGAT expression, consistent with the findings of Ostojic et al., who demonstrated that exogenous GAA suppresses endogenous GAA synthesis via feedback regulation (23). This feedback regulation may reduce endogenous GAA synthesis and potentially spare arginine for other metabolic pathways. The increase in GAMT expression may reflect an enhanced capacity for local conversion of GAA to creatine.

The unchanged CK may be due to much lower CK expression in intestine than in muscle tissue. This observation aligns with reports that GAA-mediated metabolic effects are primarily systemic and that CK expression is most prominent in skeletal muscle and neural tissues (24–26).

GAA concentration was higher in the duodenal mucosa than in the jejunum. Because mucosal concentration reflects the balance among luminal exposure, absorption, and local metabolism, it cannot be used alone to identify the primary absorption site (27–31). Future studies combining isotope tracing and intestinal flux measurements are needed to clarify segment-specific GAA absorption and metabolism in sheep.

### 4.3 GAA-regulated nutrient transport and portal plasma nutrient concentrations

RPGAA was associated with higher jejunal mRNA abundance of SLC6A6 and SLC6A8. The higher expression of SLC6A6 and SLC6A8 in the jejunum suggests an increased capacity for transport of GAA- or creatine-related substrates, although the exact regulatory mechanism requires further study (32, 33). The regulatory mechanisms underlying these transcriptional changes warrant further investigation.

### 4.4 Portal nutrient flow and hepatic IGF-1 synthesis

Insulin-like growth factor-1 (IGF-1) serves as a central endocrine mediator associated with nutrient availability to growth regulation and is predominantly synthesized in the liver in response to nutritional cues (34). In the present study, hepatic IGF-1 mRNA expression and IGF-1 concentrations in portal plasma and liver homogenates were all significantly higher in the RPGAA group than in the control and GAA groups. These variables changed in the same direction as portal glucose and selected amino acid concentrations, suggesting an association between portal nutrient status and hepatic IGF-1 indices. (35) Enhanced post-ruminal nutrient supply and improved blood metabolite profiles have been linked to better growth and metabolic status in cattle. Similar findings were reported by Bower et al., who demonstrated that direct perfusion of glucose and amino acids stimulates IGF-1 gene expression in hepatocytes (36). RPGAA-induced improvements in growth performance are associated with changes in intestinal nutrient transport and hepatic IGF-1 expression. Previous studies in cattle have likewise shown that GAA supplementation can coincide with changes in nutrient digestibility and systemic metabolic profiles, but the mechanistic link to hepatic IGF-1 regulation remains to be defined (37).

### 4.5 Serum biochemical indicators and safety profile

Serum biochemical analysis indicates that RPGAA supplementation was not accompanied by adverse changes in the measured clinical chemistry variables. Elevated serum glucose and albumin concentrations in the RPGAA group may indicate improved intestinal absorption and enhanced hepatic synthetic capacity. The decrease in serum total cholesterol may indicate altered lipid metabolism, although tissue fat deposition was not measured in the present study. The lower serum bilirubin concentration suggests that GAA supplementation did not impair hepatic function; however, antioxidant status was not directly evaluated (37). Importantly, all key indicators of liver and kidney function remained within normal ranges, with no adverse intergroup differences, suggesting no adverse effects on the measured liver- and kidney-related biomarkers at the tested dose.

### 4.6 Limitations and future directions

Although the present results are consistent with a gut–liver axis involvement in the response to RPGAA, several limitations should be considered.

1. The ruminal degradability and intestinal release efficiency of RPGAA were not verified experimentally, which is the key premise of this study.
2. The present study did not assess phosphorylation levels of key signaling proteins within the intestinal mTORC1 or hepatic PI3K–Akt pathways.
3. Portal blood samples were collected after overnight fasting, which may reflect basal metabolism rather than post-prandial nutrient absorption flux.
4. The exact intestinal absorption site of GAA was not confirmed due to the limitation of detection methods.

## 5 Conclusion

Dietary supplementation with rumen-protected guanidinoacetic acid is associated with improved growth performance in Hu sheep, and these beneficial effects are related to increased GAA and creatine concentrations in the jejunal mucosa, higher portal nutrient concentrations, and elevated hepatic IGF-1 expression. These findings suggest that RPGAA-induced growth promotion is associated with coordinated changes in intestinal nutrient transport and hepatic growth-related metabolism. Further studies are needed to verify the post-ruminal availability of RPGAA and to clarify the underlying signaling pathways in ruminants.

## Data Availability

The original contributions presented in the study are included in the article, further inquiries can be directed to the corresponding authors.
